# Global Spatiotemporal Gait Performance and Ambulation Capacity After Total Knee Arthroplasty: A Longitudinal Comparison of Fixed-Bearing and Mobile-Bearing Prostheses

**DOI:** 10.3390/jfmk11040384

**Published:** 2026-09-25

**Authors:** Andrei Machado Viegas da Trindade, Leonardo Pinheiro Rezende, Helder Rocha da Silva Araújo, Junichiro Sado Junior, Halley Paranhos Júnior, Karla Cristina Naves de Carvalho, Marcos Henrique Alves Resende, Heren Nepomuceno Costa Paixão, Juliana Oliveira Hassel Mendes, Veronica Cimolin, Rodolfo Borges Parreira, Claudia Santos Oliveira

**Affiliations:** 1 Faculty of Medicine, University Evangelica of Goiás, Anápolis 75083-515, Brazil; andrei.trindade@einstein.br (A.M.V.d.T.); medkarcri@yahoo.com.br (K.C.N.d.C.); heren.paixao@docente.unievangelica.edu.br (H.N.C.P.); juohmendes@yahoo.com.br (J.O.H.M.); 2 Department of Orthopedics Surgery, Municipal Hospital of Aparecida de Goiânia (HMAP), Aparecida de Goiânia 74936-600, Brazil; junichiro.sado@einstein.br (J.S.J.); drmharcosresende@gmail.com (M.H.A.R.); 3 School of Medical Sciences, Santa Casa de São Paulo, São Paulo 01221-020, Brazil; 4 Graduate Program in Health Sciences, Federal University of Goiás (UFG), Goiânia 74605-050, Brazil; drhelderrocha@hotmail.com; 5 Hospital Israelita Albert Einstein, São Paulo 01221-020, Brazil; 6 State Center for Rehabilitation and Readaptation Dr. Henrique Santillo (CRER), Goiânia 74653-230, Brazil; spacocontabil@terra.com.br; 7 Human Movement and Rehabilitation Post Graduation Program, Evangelical University of Goiás (UniEVANGELICA), Anápolis 75083-515, Brazil; veronica.cimolin@polimi.it (V.C.); rodolfofcmscsp@gmail.com (R.B.P.); csneuro@gmail.com (C.S.O.); 8 Department of Electronics, Information and Bioengineering, Politecnico di Milano, 20133 Milan, Italy; 9 IRCCS Istituto Auxologico Italiano, San Giuseppe Hospital, Piancavallo, 28823 Verbania, Italy

**Keywords:** 2-Minute Walk Test, fixed-bearing, gait analysis, inertial measurement unit, mobile-bearing, rotating-platform, spatiotemporal parameters, total knee arthroplasty

## Abstract

**Background**: The choice between fixed-bearing (FB) and mobile-bearing (MB) prostheses in total knee arthroplasty (TKA) remains controversial, and longitudinal evidence based on objective instrumented assessment is limited. **Objectives**: To compare global spatiotemporal gait performance and ambulation capacity across three postoperative assessments in patients receiving FB versus MB/rotating-platform implants. **Methods**: In this retrospective, longitudinal, observational cohort study, patients who had undergone primary unilateral TKA were assessed at up to three postoperative visits approximately 12 months apart, the first at a median of 12 months after surgery. Gait speed, cadence, stride length, a step-time symmetry index and a gait quality index were obtained from a single lumbar-mounted inertial measurement unit during steady-state level walking, and ambulation capacity was measured with the 2-Minute Walk Test (2MWT). Linear mixed-effects models with a random intercept per patient, adjusted for age and sex, were fitted for all six outcomes, with time specified both as a linear term and, in a sensitivity analysis, as a categorical factor yielding estimated marginal means and between-group contrasts at each visit. **Results**: A total of 105 observations from 54 patients were analysed (29 FB, 25 MB/PS-RP). Groups were comparable at the first assessment on age, sex, body mass index, WOMAC score and all gait outcomes. No between-group difference reached statistical significance for any outcome, in either the linear or the categorical specification; all 18 visit-specific contrasts included the null value. Within the FB group, gait speed, stride length, the symmetry index, the gait quality index and 2MWT distance decreased significantly between the second and third assessments, a change based on six patients. **Conclusions**: Under the conditions of this study, no significant differences in global spatiotemporal gait performance or ambulation capacity were detected between FB and MB/rotating-platform prostheses. Because the study was not designed or powered as an equivalence trial, lacks preoperative and joint-level kinematic data, and cannot separate bearing design from the surgical approach with which it was collinear, these findings do not establish that the two designs perform equivalently.

## 1. Introduction

Knee osteoarthritis (OA) is a leading cause of pain and functional disability worldwide, and the prevalence of symptomatic knee OA and the demand for total knee arthroplasty (TKA) continue to increase [1,2,3,4]. TKA relieves pain and restores function in patients with end-stage OA; however, residual gait deficits are common and may influence patient satisfaction and functional independence [5,6,7,8].

The success of TKA depends on multiple factors, including implant design. A persistent controversy is the choice between fixed-bearing (FB) and mobile-bearing (MB) tibial inserts [9]. The FB design involves a biomechanical compromise: greater conformity may reduce contact stress but increase torque transmission and the theoretical risk of loosening, whereas lower conformity may reduce torque but increase contact stress and wear [10].

MB designs were introduced to address this compromise by allowing the polyethylene insert to rotate or translate, thereby decoupling axial rotation from constraint. In theory, this design may promote more physiological kinematics, reduce contact stress, and improve functional outcomes [11]. Self-alignment of the mobile platform may also increase contact area and reduce stress peaks [12]. The clinical evidence, however, has not borne this out, and it is important to be specific about which outcomes have been examined. Meta-analyses and randomised trials comparing the two designs have reported no consistent superiority of MB over FB for patient-reported function and pain, range of motion, joint awareness, crepitus, or implant survivorship [13,14]. These are predominantly patient-reported and clinical endpoints; they do not directly address whether the two designs produce measurably different walking mechanics.

Instrumented gait analysis addresses that question directly. Early three-dimensional gait studies established that TKA design influences walking and stair-climbing mechanics, particularly the knee flexion–extension pattern and the quadriceps-avoidance gait that accompanies it [15], and a systematic review of gait after knee replacement subsequently confirmed that patients retain slower walking speed, reduced stride length and diminished knee excursion relative to healthy controls even when clinical scores are satisfactory [16]. Laboratory studies comparing bearing designs have concentrated on joint-level and transverse-plane variables, where the theoretical advantage of a rotating platform should be expressed: fluoroscopic and kinematic work has documented greater axial rotation in some MB designs, but without a corresponding change in the global walking pattern [11,17]. Studies that have compared spatiotemporal descriptors specifically—walking speed, cadence, stride length and interlimb symmetry—have generally found the two designs indistinguishable, although sample sizes have been modest and follow-up mostly shorter than two years [18,19].

Wearable inertial measurement units (IMUs) have made this kind of assessment feasible outside the gait laboratory. A single trunk-mounted sensor yields walking speed, cadence, stride length and temporal symmetry indices with documented accuracy against optoelectronic and instrumented-walkway reference systems [20,21,22,23,24], and systematic reviews in the TKA population identify these global spatiotemporal descriptors as the parameters most consistently reported and most responsive to postoperative recovery [25,26,27]. It is important to recognise what this configuration does and does not measure: it quantifies how the patient walks overall, not the conformity, contact mechanics or rotational behaviour of the implant itself. The 2-Minute Walk Test (2MWT) complements instrumented analysis by capturing sustained ambulation capacity in a single, clinically interpretable distance [28,29,30,31,32]. Longitudinal studies applying instrumented assessment to the comparison of bearing designs nonetheless remain scarce.

Therefore, the objective of this study was to compare global spatiotemporal gait performance and ambulation capacity, measured with a wearable inertial sensor and the 2MWT, between patients who underwent TKA with FB versus MB/rotating-platform implants, across three postoperative assessments. Two research questions were addressed: whether global spatiotemporal gait performance and ambulation capacity differ between the two bearing designs across the postoperative period, and how these measures change over successive assessments within each group. Neither question was framed as a directional hypothesis, because the design permits a difference to be identified but cannot establish its absence. The study was designed to characterise global walking performance and was not intended, and is not able, to evaluate implant-level kinematics, contact mechanics or load distribution.

## 2. Materials and Methods

### 2.1. Study Design and Setting

A retrospective, longitudinal, observational cohort study was conducted and is reported in accordance with the Strengthening the Reporting of Observational Studies in Epidemiology (STROBE) statement [33]. Data were obtained from patients who underwent primary unilateral TKA between 2019 and 2022 at the State Center for Rehabilitation and Readaptation Dr. Henrique Santillo (CRER), a tertiary referral centre in Goiania, Goias, Brazil, and who completed functional assessments at one or more postoperative follow-up visits. Because the data were collected during routine rehabilitation review rather than at fixed anniversaries, the assessments are designated the first (T1), second (T2) and third (T3) postoperative assessments throughout this article. The first assessment was performed at a median of 12 months after surgery (interquartile range 12–18 months; range 7–21 months), and subsequent assessments followed at intervals of approximately 12 months. No preoperative assessment was available for any patient. All assessments were performed at the same centre by the same trained evaluators.

### 2.2. Participants, Implants and Allocation

Consecutive patients with end-stage primary knee OA who underwent primary unilateral TKA were eligible. Patients were excluded if they underwent bilateral or unicompartmental arthroplasty or revision surgery, or if they had postoperative infection or another clinical condition capable of substantially affecting gait. All procedures were primary unilateral TKAs performed at a single centre by the institutional arthroplasty team. Both designs were posterior-stabilised and therefore entailed sacrifice of the posterior cruciate ligament, so the groups differed in the tibial bearing rather than in ligament management: the FB group received a posterior-stabilised implant with a fixed tibial plateau, whereas the MB/PS-RP group received a congruent rotating-platform implant.

Implant allocation was not randomised and followed established surgeon practice patterns rather than patient-level clinical criteria. Each operating surgeon in the team used one bearing design as their habitual choice, and the design a given patient received was therefore determined by which surgeon performed the procedure, which in turn depended on operating-list scheduling. The records contain no indication that deformity severity, intraoperative stability, ligament balance or other patient characteristics were used to select between the two designs. This allocation mechanism has a consequence that must be stated explicitly: the surgical approach was almost perfectly collinear with bearing type, because each surgeon used their customary arthrotomy. A transquadricipital approach was used in 52 of the 53 fixed-bearing observations, and a subvastus approach in all 52 mobile-bearing observations. Any difference between the groups in this cohort is therefore attributable to the combination of bearing design and surgical approach, and the two cannot be separated. The clinical records available for this retrospective analysis did not document implant manufacturer or model, articular conformity, polyethylene insert thickness, patellar resurfacing, limb-alignment strategy, soft-tissue release performed, preoperative deformity pattern or its magnitude, Kellgren–Lawrence grade, contralateral knee status, comorbidity burden, involvement of other joints, or the intensity of postoperative rehabilitation. These variables could therefore neither be characterised nor entered into the analysis, and their potential contribution to residual confounding is addressed in Section 4. No a priori sample size calculation was performed, because of the retrospective design.

### 2.3. Procedures and Outcomes

Functional assessments comprised instrumented gait analysis and a field test of ambulation capacity. Both were performed at the same visit, in the same order, with the instrumented walking trial first and a seated rest of at least five minutes before the walk test. The layout of both assessments is shown schematically in Figure 1, and the two components are described below.

#### 2.3.1. Instrumented Gait Analysis

Gait was assessed with a BTS G-Walk wearable inertial sensor (BTS Bioengineering, Milan, Italy) secured over the lumbar region at approximately the level of the fifth lumbar vertebra, close to the body centre of mass, using the manufacturer’s semi-elastic belt. The unit combines a triaxial accelerometer (+/−16 g), a triaxial gyroscope (+/−2000 degrees/s) and a triaxial magnetometer, and transmits data by Bluetooth at a sampling rate of 100 Hz. Signals were acquired and processed with the manufacturer’s G-Studio software (Version 3.3.22, BTS Bioengineering, Milan, Italy) using the default Walk+ protocol settings, without user modification of filter or event-detection parameters. Within that protocol, raw acceleration and angular-velocity signals are band-pass filtered to attenuate high-frequency noise and low-frequency drift, and initial and final foot contacts are identified from the anteroposterior and vertical acceleration components; gait speed, cadence, stride length, the step-time symmetry index and the gait quality index are then derived from the detected events. The underlying single-accelerometer algorithm was originally validated against three-dimensional optoelectronic gait analysis by Bugane et al. [20], and the commercial implementation has since been compared with optoelectronic and instrumented-walkway reference systems in healthy adults [21,22], in active older adults [23] and in patients with Parkinson disease [24]. The precise software build number was not recorded in the source dataset, and test–retest reliability was not formally re-established in the present cohort; published validation in comparable adult populations was therefore relied upon. The limits of this evidence for asymmetry-related outcomes are addressed in Section 4.

Patients walked along a level, unobstructed indoor corridor 30 m long and approximately 2 m wide, with a smooth vinyl floor and no doorways or obstacles along the measured section (Figure 1a). They were instructed to walk straight ahead to the far end of the corridor in a single pass and were not required to turn at any point during the recorded trial. Walking speed was self-selected and standardised by verbal instruction rather than by a perceived-exertion scale: each patient was asked, in a fixed form of words, to walk at the comfortable pace they would use to cross a level street without hurrying, and no pacing, encouragement or feedback was given during the trial. To restrict the analysis to steady-state locomotion, the acceleration and deceleration phases at the start and end of each walking bout were discarded, and at least ten consecutive steady-state strides were required for a trial to be retained.

The step-time symmetry index quantified interlimb temporal balance and was expressed as a percentage in which 100% denotes perfect right-left symmetry. It was computed from the mean step times of the two limbs as follows:

SI (%) = [1 − |t_R_ − t_L_|/(t_R_ + t_L_)] × 100 (1)

where t_R_ and t_L_ are the mean step times of the right and left limbs, respectively. Values approaching 100% indicate progressively smaller differences between the step times of the two limbs, whereas lower values reflect greater temporal asymmetry. The gait quality index reported by the device is a separate, composite descriptor derived by the manufacturer’s software from the regularity and reproducibility of the acceleration pattern at the trunk; it is reported here alongside the symmetry index and is not interchangeable with it.

Stride length is reported throughout this article as the distance covered by one complete gait cycle, that is, between two successive contacts of the same foot, which is the quantity the device outputs. It is approximately twice the step length, and the two terms are not used interchangeably.

#### 2.3.2. Two-Minute Walk Test

Functional ambulation capacity was assessed with the 2MWT [30]. Patients were instructed to cover as much ground as possible in two minutes at a brisk but safe self-selected pace along a flat 30 m course delimited by two cones, turning 180 degrees around each cone at the ends of the course, and the total distance walked was recorded in metres (Figure 1b). Standardised verbal instructions were given before the test and standardised encouragement was provided at fixed intervals. Participants wore their usual footwear and were allowed to use their habitual walking aid when one was normally required, with the same device used at every assessment for a given patient. The 2MWT distance is reported as the raw distance walked. No adjustment or correction was applied to it, and no velocity was derived from it for analysis; the relationship between this distance and the steady-state corridor speed is considered qualitatively in Section 4.

### 2.4. Ethical Considerations

The study was conducted in accordance with the Declaration of Helsinki and was approved by the Research Ethics Committee of Universidade Evangélica de Goiás (UniEVANGÉLICA), Anápolis, Goiás Evangélica de Goias (UniEVANGELICA), Anapolis, Goias, Brazil (CAAE 52052421.9.0000.5076; approval no. 6,775,127, granted on 19 April 2024), under the terms of Resolution 466/2012 of the Brazilian National Health Council. The functional assessments were performed within an approved research protocol for which participants provided written informed consent, and the present analysis of the resulting data was covered by the approved amendment authorising the longitudinal follow-up. All records were anonymised before analysis.

### 2.5. Statistical Analysis

Analyses were performed in Python (version 3.13) using the statsmodels library, with statistical significance set at *p* < 0.05. Comparisons between prosthesis groups at the first postoperative assessment were made using independent-samples t-tests or Mann–Whitney U tests for continuous variables, according to the Shapiro–Wilk test of distributional normality, and chi-square or Fisher exact tests for categorical variables. The longitudinal effect of prosthesis type was examined with linear mixed-effects models that included prosthesis type, time, and the time-by-prosthesis interaction as fixed effects, a random intercept for each patient, and adjustment for age and sex. Age and sex were the only covariates available for all participants; the variables listed in Section 2.2 were not recorded in the source dataset and could not be entered into the models. The models used all available observations under a missing-at-random assumption, which accommodates the unbalanced structure produced by the differing number of assessments contributed by each patient. The plausibility of that assumption is considered in Section 4 in the light of the participant flow reported in Section 3.1.

All six outcomes—gait speed, cadence, stride length, the step-time symmetry index, the gait quality index and the 2MWT distance—were modelled inferentially, so that every statistical statement in this article rests on a fitted model rather than on a descriptive comparison. Gait speed, cadence and stride length are mathematically related, since speed is the product of cadence and step length; they are modelled separately as descriptors of how a given walking speed is achieved, and are interpreted jointly rather than as independent effects. Two specifications of time were fitted for each outcome. In the primary specification, time was entered as a linear term across the three assessments, summarising the average trend. Because the descriptive pattern was not monotonic, a prespecified sensitivity analysis re-fitted every model with time as a categorical factor, from which estimated marginal means with 95% confidence intervals were computed at each assessment, together with between-group contrasts at each assessment and within-group contrasts between assessments. Marginal means were evaluated at the observed mean age and sex distribution of the analysed sample. Given the small number of patients assessed at T3, the categorical estimates at that visit are imprecise, and this is reflected in the width of the reported confidence intervals. No adjustment for multiple comparisons was applied; the contrasts are reported as pre-specified descriptive quantities with their confidence intervals rather than as independent hypothesis tests, and are interpreted accordingly. No equivalence or non-inferiority margin was prespecified, so the analysis can identify a difference but cannot establish its absence.

## 3. Results

### 3.1. Participant Characteristics and Flow

Fifty-seven patients who had undergone primary unilateral TKA were identified in the institutional rehabilitation records. Three of them had no valid instrumented gait assessment and were not analysed, leaving 54 patients who contributed 105 valid observations across the three postoperative assessments. The analysed cohort comprised 29 patients with an FB implant and 25 with an MB/PS-RP implant. At the first assessment, 54 patients were evaluated (29 FB, 25 MB/PS-RP); at the second, 36 (18 FB, 18 MB/PS-RP); and at the third, 15 (6 FB, 9 MB/PS-RP). Demographic, anthropometric and clinical characteristics at that assessment are summarised in Table 1. No statistically significant between-group difference was observed at the first assessment for age, sex, body weight, height, body mass index, WOMAC total score, operated side, 2MWT distance, gait speed, cadence, stride length, the step-time symmetry index or the gait quality index, indicating that the groups were comparable on all measured variables at the start of follow-up. Comparability at the first postoperative assessment does not establish that the groups were comparable before surgery, a question which cannot be determined from these data.

The longitudinal structure of the dataset is shown in Figure 2. Of the 54 analysed patients, 15 contributed a single assessment, 27 contributed two and 12 contributed all three; 12 patients attended all three consecutive visits and a further three attended the first and third but not the second. Eighteen patients (11 FB, 7 MB/PS-RP) had no second assessment and 39 (23 FB, 16 MB/PS-RP) had no third assessment; of the 36 patients assessed at the second visit, 24 (13 FB, 11 MB/PS-RP) did not go on to a third. Two mechanisms account for these missing observations. The first is administrative censoring: because the patients were operated on between 2019 and 2022 and the assessments were drawn from routine rehabilitation review, those treated more recently had not yet reached the later assessment windows when the data were collected, so the observation could not exist. The second is non-attendance at a scheduled review, for which the records do not document a reason. The two mechanisms cannot be distinguished retrospectively for every individual patient, and the source records do not contain the information needed to do so. No patient was excluded after enrolment because of revision surgery or periprosthetic infection, so failure of the arthroplasty does not account for the attrition. All available observations were retained in the mixed-effects models. The implication of this structure for the missing-at-random assumption is examined in Section 4.

### 3.2. Longitudinal Outcomes of Functional Performance and Gait

Descriptive statistics for all six outcomes at each assessment, stratified by prosthesis type, are presented in Table 2, and the trajectories are shown in Figure 3 for the 2MWT and Figure 4 for the instrumented gait parameters. Both groups showed higher mean 2MWT distance at the second assessment than at the first (FB 75.2 to 89.1 m; MB/PS-RP 73.0 to 87.0 m). At the third assessment, the mean 2MWT distance was lower in the FB group (64.2 m) than in the MB/PS-RP group (84.3 m), and the same ordering was present for gait speed, cadence, stride length, the symmetry index and the gait quality index. Only six FB patients and nine MB/PS-RP patients were assessed at that visit, and the dispersion at the third assessment was correspondingly wide, as the error bars in Figure 3 and Figure 4 show.

### 3.3. Effect of Prosthesis Type over Time

The linear-time mixed-effects models adjusted for age and sex are summarised in Table 3. No statistically significant main effect of prosthesis type was found for any of the six outcomes: gait speed (*p* = 0.478), cadence (*p* = 0.889), stride length (*p* = 0.334), step-time symmetry index (*p* = 0.919), gait quality index (*p* = 0.778) or 2MWT distance (*p* = 0.584). Neither the main effect of time nor the time-by-prosthesis interaction reached significance for any outcome, indicating that the average trend across the follow-up period did not differ significantly between the FB and MB/PS-RP groups.

### 3.4. Sensitivity Analysis with Time as a Categorical Factor

Because the descriptive trajectories were not monotonic, the models were re-fitted with time as a categorical factor. Estimated marginal means with 95% confidence intervals at each assessment are reported in Table 4, and the between-group contrasts in Table 5. All 18 visit-specific between-group contrasts had confidence intervals that included the null value. The largest estimated differences occurred at the third assessment and all favoured the MB/PS-RP group: 2MWT distance 16.1 m (95% CI −2.8 to 35.0; *p* = 0.096), gait speed 0.17 m/s (95% CI −0.05 to 0.39; *p* = 0.123) and cadence 7.9 steps/min (95% CI −2.1 to 17.8; *p* = 0.122). The confidence intervals at this visit are wide enough to encompass both a clinically important advantage for the MB/PS-RP design and no difference at all, so they neither establish nor exclude a late divergence between designs.

The within-group contrasts between assessments are reported in Table 6. Both groups improved significantly in 2MWT distance between the first and second assessments (FB +13.6 m, 95% CI 3.8 to 23.4, *p* = 0.007; MB/PS-RP +13.8 m, 95% CI 3.8 to 23.8, *p* = 0.007). Between the second and third assessments the FB group declined significantly in 2MWT distance (−22.4 m, 95% CI −38.3 to −6.5, *p* = 0.006), gait speed (−0.23 m/s, 95% CI −0.41 to −0.04, *p* = 0.016), cadence (−8.6 steps/min, 95% CI −16.6 to −0.6, *p* = 0.036), stride length (−0.22 m, 95% CI −0.38 to −0.06, *p* = 0.009) and the gait quality index (−4.4 percentage points, 95% CI −8.0 to −0.7, *p* = 0.018), and relative to the first assessment, the FB group was significantly lower at the third assessment in gait speed, stride length, the step-time symmetry index and the gait quality index. No within-group change reached significance in the MB/PS-RP group between the second and third assessments. These within-group changes in the FB group rest on the six patients assessed at T3 and are reported as observations within this cohort rather than as evidence of a design-related effect; the corresponding between-group contrasts, which are the relevant comparison for the study question, were not significant.

## 4. Discussion

In this cohort, no statistically significant difference in global spatiotemporal gait performance or ambulation capacity was detected between patients who received FB and those who received MB/PS-RP implants, at any of three postoperative assessments, under the two specifications of time. The correct reading of this result is narrow and worth stating precisely: under the conditions of this study, with this sample size and this measurement approach, no difference was detected. That is not the same as having shown that the two designs perform equivalently. The study prespecified no equivalence or non-inferiority margin, the sample was modest and non-randomised, a substantial proportion of observations were missing at the later assessments, and no preoperative data were available, so the absence of a detected difference is uninformative about whether a difference of clinically relevant size exists.

A second limitation of inference is more specific to this cohort and bears directly on how the result should be attributed. Because each surgeon used both a habitual bearing design and a habitual arthrotomy, surgical approach was almost perfectly collinear with bearing type: essentially all FB procedures used a transquadricipital approach and all MB/PS-RP procedures a subvastus approach. The two factors cannot be statistically separated in these data. The subvastus approach spares the extensor mechanism and has been associated with earlier recovery of quadriceps function, so it is a plausible independent influence on walking performance. Any finding in this cohort therefore concerns the combination of bearing design and approach, not bearing design alone, and the absence of a detected difference could in principle reflect two genuine effects of opposite sign that cancel. Beyond this, the source records did not document ligament balancing or the distraction force applied during balancing, limb-alignment strategy, implant manufacturer or conformity, polyethylene thickness, patellar resurfacing, soft-tissue release, preoperative deformity, Kellgren–Lawrence grade, contralateral knee status, comorbidity burden, or rehabilitation intensity. Each of these can influence postoperative stability, kinematics and gait independently of the tibial bearing. Recent evidence has quantified the distraction forces commonly applied during tension-controlled ligament-balanced TKA and has highlighted the potential relevance of individualised balancing to postoperative joint behaviour [34]; the present dataset contains no information on this aspect of the procedure. These omissions constitute substantial confounding by indication and practice pattern, and they mean the outcomes observed here cannot be attributed specifically to FB versus MB design.

A third consideration concerns what the measurement can detect. The theoretical arguments for a rotating platform are made at the level of the joint: articular conformity, contact stress and its distribution, transverse-plane rotation and the constraint imposed on it. A single trunk-mounted IMU does not measure any of these. It measures how the whole body progresses along a corridor—speed, cadence, stride length and the temporal regularity of successive steps—and these global descriptors can remain unchanged while joint-level mechanics differ substantially. Biomechanical work has documented greater axial rotation in some MB designs [11], and differences in loading across levels of constraint [35], without corresponding differences in walking speed. The systematic review literature makes the same point from the clinical side: abnormal gait patterns persist after TKA even when patient-reported outcomes are satisfactory [16], so global performance is an imperfect proxy for implant mechanics. The present finding should therefore be read as evidence about global walking performance and ambulation capacity, and not as evidence that the kinematic differences attributed to rotating-platform designs are absent or functionally irrelevant. Testing that claim requires instrumentation this study did not use.

The measurement limits are sharpest for the asymmetry-related outcome. Single trunk-mounted configurations have been validated against reference systems for walking speed, cadence and stride length, with intraclass correlation coefficients typically above 0.8 for these parameters [20,21,22,23,24]; validation is markedly weaker for variables that require limb-specific event detection, and one recent concurrent-validity study of this sensor configuration reported good agreement for cadence, speed and stride length but poor agreement for gait-phase parameters in the same participants [22]. Methods for deriving asymmetry from a single low-back accelerometer have been developed and validated chiefly in post-stroke cohorts [36,37], where interlimb differences are large; no published study has established the accuracy of this device’s symmetry index against a reference standard in a TKA or comparable arthroplasty population, where asymmetries are considerably smaller. The step-time symmetry index and the gait quality index reported here should accordingly be interpreted with more caution than the speed, cadence and stride-length results, and the absence of a detected between-group difference in symmetry is correspondingly weaker evidence. Establishing the measurement properties of trunk-derived asymmetry indices in arthroplasty populations is a prerequisite for using them to compare implant designs.

The recovery pattern was not monotonic: performance was higher at the second assessment than at the first in both groups, and lower at the third in the FB group. The linear-time models summarise the average trend and cannot represent this shape, which is why the categorical sensitivity analysis was fitted; that analysis localised the significant changes to the second-to-third interval within the FB group while showing no significant between-group difference at any visit. Interpretation of the late decline requires restraint. Six FB patients were assessed at the third visit. At that sample size, ordinary between-subject variability, or a small number of interval events not captured in the dataset, is sufficient to produce a group-level decline of the observed magnitude. Patients who underwent revision or developed infection were excluded by design, so procedure-related failure does not explain it. Contralateral knee OA is a plausible contributor, since knee OA is frequently bilateral and improved function of the operated limb may increase demand on the other side; contralateral pain exacerbation after TKA has been reported to impair functional activity [38], and the present dataset records neither contralateral symptoms nor radiographic status. Progression of OA in other joints, an unrelated musculoskeletal injury, a fall, or intercurrent illness would have the same effect, and are equally unrecorded. The third-assessment findings are hypothesis-generating and should not be read as evidence of late divergence between designs, particularly as the between-group contrasts at that visit were not significant.

The participant flow has a further implication for the models. Eighteen patients had no second assessment and 39 no third, and part of this attrition is administrative censoring, which is unrelated to the patient’s functional status and therefore compatible with the missing-at-random assumption underlying the mixed-effects models. The remainder is non-attendance at a scheduled review, for which the records give no reason. If patients who were walking poorly were more likely to miss a review, or conversely if those who had recovered well saw less need to attend, the missingness would depend on the unobserved outcome and the assumption would be violated in a direction that cannot be determined from these data. This is a material limitation of the longitudinal estimates, particularly at the third assessment, and it cannot be resolved retrospectively.

The 2MWT distances were lower than the product of the instrumented gait speed and the test duration. This follows from the different conditions of the two measurements rather than from any inconsistency between instruments. The inertial sensor reports steady-state speed during a single straight-line pass with the acceleration and deceleration phases removed, whereas the 2MWT was performed over a 30 m course traversed repeatedly with a 180-degree turn at each end; deceleration before each turn and reacceleration afterwards reduce the average velocity sustained over the two minutes. No correction for this effect was applied to the 2MWT distance, which is reported as the raw distance walked, and no velocity was derived from it for any analysis. Interpreting the magnitude of the observed changes remains difficult because minimally clinically important differences for instrumented gait parameters have not been established in the TKA population. For the 2MWT, the within-group improvement of approximately 14 m between the first and second assessments exceeds the minimal detectable change reported for this test after knee arthroplasty, whereas the between-group differences were smaller than the reported measurement variability [29,31,32], which argues against a clinically meaningful divergence between designs within the period studied.

These considerations point to a wider methodological issue. Systematic reviews of wearable gait analysis consistently report heterogeneity in sensor number and placement, sampling and filtering choices, event-detection algorithms and walking protocols, which complicates comparison across studies and constrains pooling of results [25,26,27]. Single-point trunk-mounted configurations are attractive for clinical use because they are quick to apply and well tolerated, and their accuracy is greatest for the global spatiotemporal descriptors that dominate the TKA gait literature. The parameters selected here therefore correspond to those with the strongest validation support for this sensor configuration and this population, and the acquisition and processing conditions have been reported above in sufficient detail to be reproduced. Convergence on standardised acquisition and reporting protocols, and derivation of clinically important difference thresholds for instrumented parameters, would materially improve the comparability of studies of implant design.

The strengths of this study are the use of objective instrumented measurement rather than patient-reported outcomes alone, the longitudinal structure spanning three assessments, the single-centre setting with consistent assessment procedures and evaluators, and the inferential treatment of all six outcomes with a prespecified sensitivity analysis for the shape of the time trend.

### 4.1. Limitations

Several limitations constrain what can be concluded, in addition to the inferential, confounding and measurement limits considered above. The sample was modest and small at the third assessment, limiting power particularly for the time-by-prosthesis interaction. Allocation was non-random and followed surgeon practice, and bearing design was collinear with surgical approach. Numerous surgical, implant-related, radiographic and clinical covariates were unavailable. No preoperative assessment existed, so neither the preoperative comparability of the groups nor the absolute magnitude of postoperative recovery can be evaluated, and the first postoperative assessment cannot serve as a substitute for a preoperative baseline. The assessment interval was defined by routine review rather than by protocol, so the timing of assessments varied between patients. A single trunk-mounted sensor cannot resolve joint-level kinematics, frontal- and transverse-plane control, stance–time asymmetry, double-support time, or turning performance, and its validation for asymmetry indices in this population is absent. Finally, established minimally clinically important differences for instrumented gait parameters are lacking in TKA, so the clinical relevance of the observed changes cannot be quantified.

### 4.2. Implications and Future Directions

What follows for practice is therefore limited and conditional. These data provide no evidence that the choice between an FB and an MB/rotating-platform design, as used by this surgical team with their respective approaches, is associated with a different trajectory of global walking performance or ambulation capacity over the period studied. They do not establish that the designs are equivalent, and they say nothing about joint-level loading, muscle strength or neuromuscular control. The study did not compare rehabilitation strategies and therefore cannot support any recommendation about how rehabilitation should be structured; whether protocols should be differentiated by implant design is a question that requires a study in which rehabilitation is the variable under investigation.

Several directions follow for future work. The immediate methodological priorities are a prospective design with a preoperative baseline, protocol-defined assessment intervals, randomised or otherwise unconfounded allocation that separates bearing design from surgical approach, prespecified equivalence or non-inferiority margins, and structured recording of the surgical, implant and clinical covariates absent here. Adequately powered randomised and preferably multicentre trials remain necessary to determine whether the two designs are functionally comparable and, if so, within what margin. Richer instrumentation would address the measurement gap: multiple IMUs on the shank and foot, or a full optoelectronic protocol, would resolve joint angles through the gait cycle and the symmetry of their recovery, and surface electromyography of the principal muscle groups during walking would characterise muscle balance and the stability of the reconstructed joint—variables that plausibly mediate any effect of bearing design and that the present protocol could not capture. Establishing minimally clinically important differences and reference validation for trunk-derived asymmetry indices in arthroplasty populations is a prerequisite for interpreting such measurements [30,39]. Looking further, the framing of the question may itself need to change. Comparisons of group mean gait parameters between implant designs treat patients as interchangeable, whereas the biomechanical response to an implant depends on individual anatomy, loading, and soft-tissue behaviour. Work in adjacent areas of musculoskeletal biomechanics illustrates a different approach: subject-specific finite-element modelling combined with machine learning has been used to tune cushioning lattice structures to an individual’s plantar pressure distribution [40] and to adapt dual-layer lattice geometry to gait-induced loading [41]. Those studies concern footwear rather than knee implants, and the analogy should not be overstated, but the methodological framework—patient-specific loading data, computational modelling, and optimisation against an individual objective—indicates a plausible route from group-level comparison of prosthesis designs toward patient-specific selection and configuration.

## 5. Conclusions

In this retrospective longitudinal cohort, no statistically significant difference in global spatiotemporal gait performance or 2-Minute Walk Test distance was detected between patients who underwent TKA with fixed-bearing and those who received mobile-bearing/rotating-platform implants, at any of three postoperative assessments, under both linear and categorical specifications of time. Both groups improved significantly in ambulation capacity between the first and second assessments. A significant decline between the second and third assessments was observed in the fixed-bearing group across several outcomes, based on six patients, while no between-group contrast reached significance at any visit. Because the study prespecified no equivalence margin, was not randomised, lacked preoperative and joint-level kinematic data, and could not separate bearing design from the surgical approach with which it was collinear, these results do not establish that the two designs perform equivalently, and these findings should not be used on their own to guide implant selection. They indicate that, within the limits of global spatiotemporal measurement over this period, no difference in walking performance was apparent—presenting a question that adequately powered prospective studies with preoperative baselines and joint-level instrumentation are needed to settle.

## Figures and Tables

**Figure 1:**
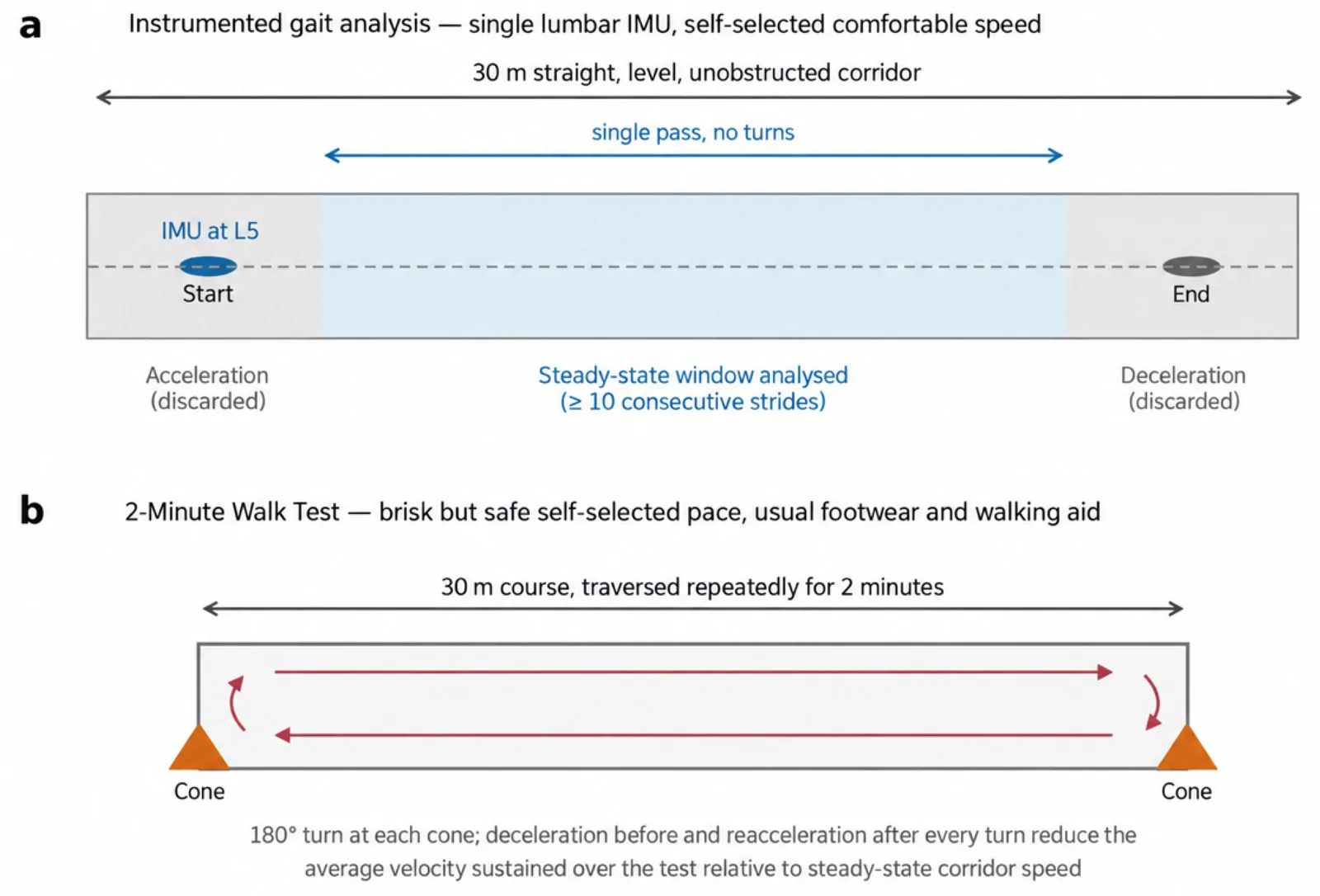
Layout of the two functional assessments. (**a**) Instrumented gait analysis: patients walked a single straight pass along a 30 m level corridor with a single inertial measurement unit secured over the lumbar region at approximately the level of the fifth lumbar vertebra; the acceleration and deceleration phases at each end were discarded and at least ten consecutive steady-state strides were retained for analysis. (**b**) 2-Minute Walk Test: patients covered as much ground as possible in two minutes along a 30 m course delimited by two cones, turning 180 degrees at each end. The deceleration and reacceleration associated with each turn reduce the average velocity sustained over the test relative to the steady-state corridor speed.

**Figure 2:**
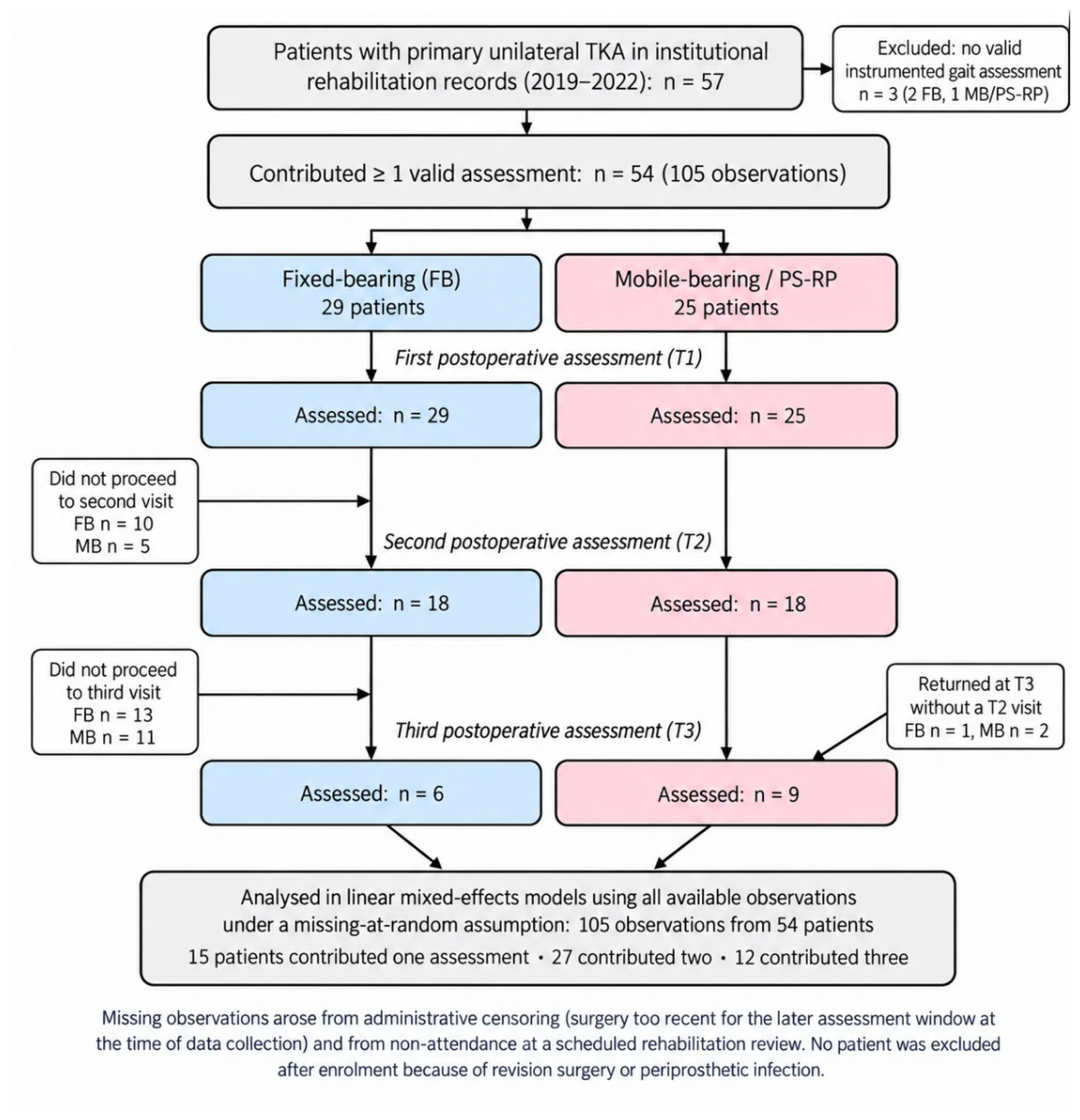
Flow of participants through the study. Of 57 patients identified in the institutional rehabilitation records, 3 had no valid instrumented gait assessment and 54 contributed 105 observations across the three postoperative assessments. The counts at the left are transition counts, that is, the patients assessed at one visit who were not assessed at the next; three patients attended the third assessment without having attended the second, so 39 patients in total (23 FB, 16 MB/PS-RP) had no third assessment. All available observations were retained in the mixed-effects models under a missing-at-random assumption. FB: fixed-bearing; MB/PS-RP: mobile-bearing/posterior-stabilised rotating-platform; T1, T2, T3: first, second and third postoperative assessments.

**Figure 3:**
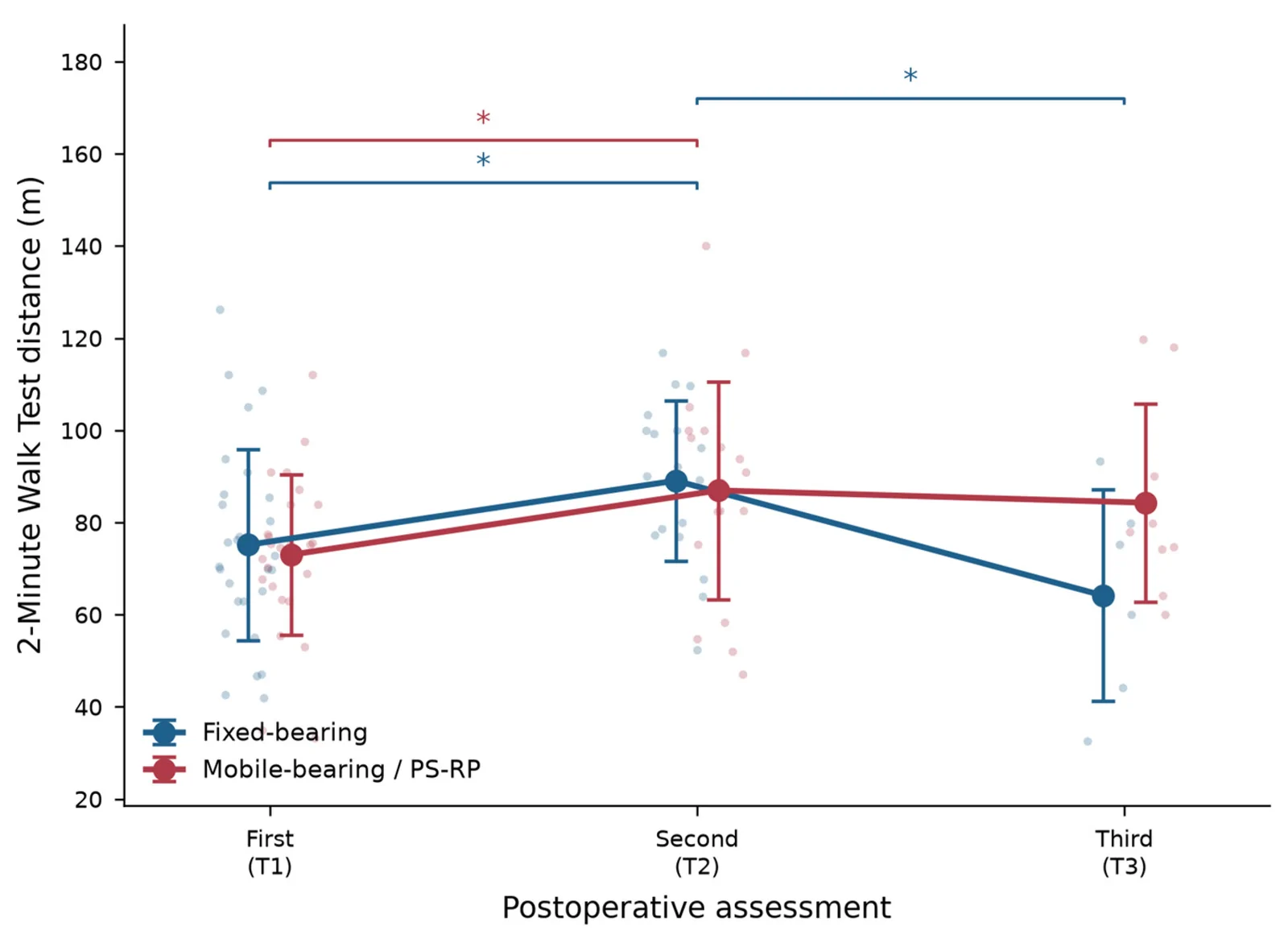
Longitudinal trajectory of the 2-Minute Walk Test distance according to prosthesis type. Filled symbols denote group means and error bars one standard deviation; small translucent symbols are the individual observations contributing to each mean, horizontally jittered for legibility. Brackets marked with an asterisk indicate within-group contrasts significant at *p* < 0.05 in the linear mixed-effects model with time as a categorical factor, adjusted for age and sex; no between-group contrast was significant at any assessment. Group means are based on 29, 18 and 6 fixed-bearing and 25, 18 and 9 mobile-bearing observations at the first, second and third assessments, respectively. FB, fixed-bearing; MB/PS-RP, mobile-bearing/posterior-stabilised rotating-platform.

**Figure 4:**
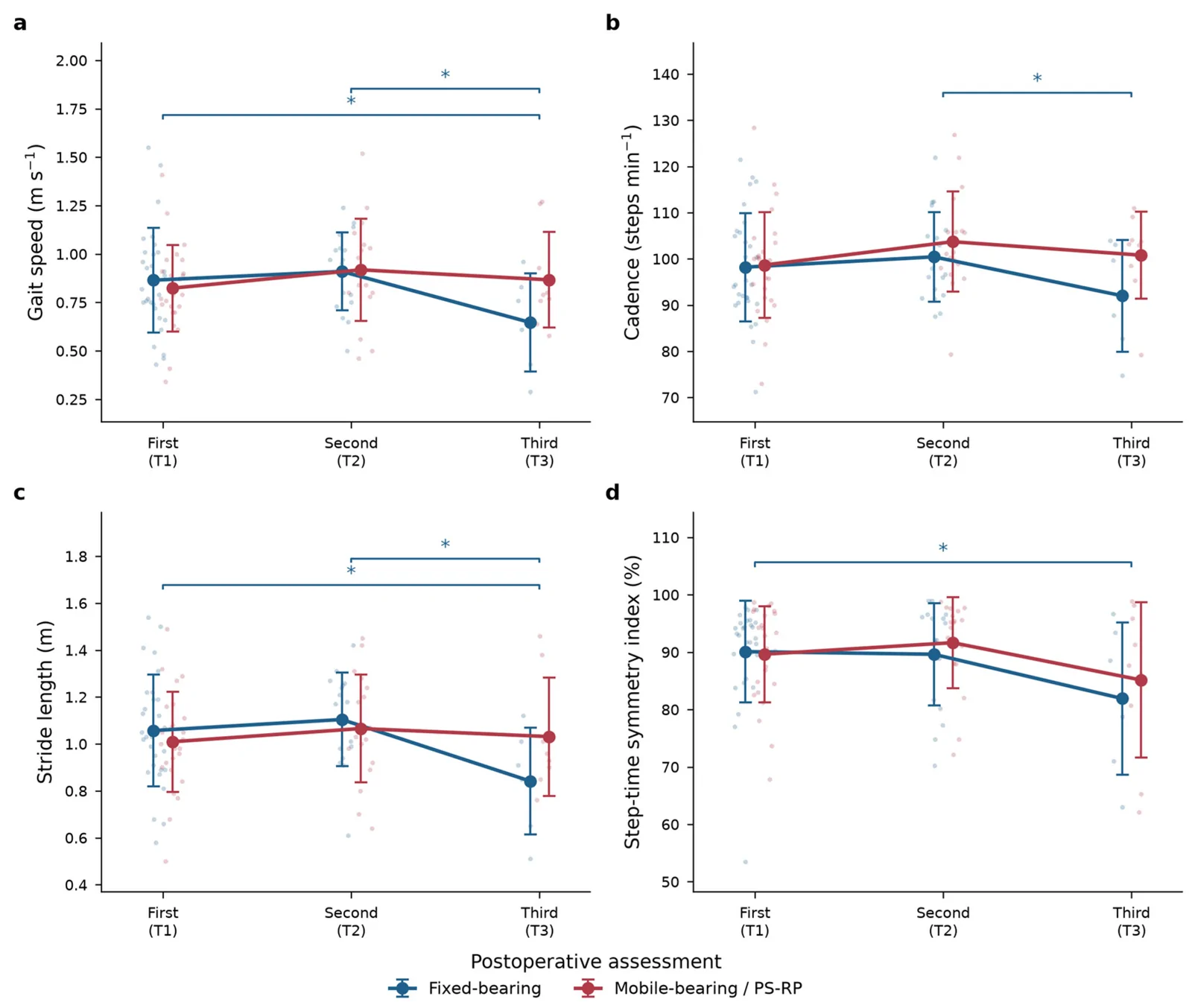
Longitudinal gait parameters obtained from the wearable inertial sensor according to prosthesis type: (**a**) gait speed; (**b**) cadence; (**c**) stride length; and (**d**) step-time symmetry index. Filled symbols denote group means and error bars one standard deviation; small translucent symbols are the individual observations contributing to each mean, horizontally jittered for legibility. Brackets marked with an asterisk indicate within-group contrasts significant at *p* < 0.05 in the linear mixed-effects model with time as a categorical factor, adjusted for age and sex; no between-group contrast was significant at any assessment. Stride length denotes the distance between two successive contacts of the same foot. FB, fixed-bearing; MB/PS-RP, mobile-bearing/posterior-stabilised rotating-platform.

**Table 1:** Demographic, anthropometric and clinical characteristics of the participants at the first postoperative assessment, stratified by prosthesis type.

Characteristic	All Patients (*n* = 54)	FB (*n* = 29)	MB/PS-RP (*n* = 25)	*p*-Value
Age (years)	67.8 (7.7)	67.8 (8.0)	67.9 (7.5)	0.954
Sex (female/male)	Female: 32/Male: 22	17/12	15/10	1.000
Weight (kg)	79.1 (13.5)	79.6 (13.3)	78.6 (14.0)	0.658
Height (m)	1.61 (0.10)	1.62 (0.10)	1.59 (0.10)	0.247
BMI (kg/m^2^)	30.7 (5.0)	30.4 (5.1)	31.2 (4.9)	0.472
WOMAC total	11.4 (11.1)	11.6 (13.0)	11.2 (8.6)	0.610
2MWT (m)	74.2 (19.1)	75.2 (20.7)	73.0 (17.4)	0.680
Gait speed (m/s)	0.85 (0.25)	0.86 (0.27)	0.82 (0.22)	0.543
Cadence (steps/min)	98.4 (11.5)	98.2 (11.7)	98.7 (11.4)	0.887
Stride length (m)	1.03 (0.23)	1.06 (0.24)	1.01 (0.21)	0.441
Step-time symmetry index (%)	89.9 (8.6)	90.1 (8.9)	89.6 (8.4)	0.993
Gait quality index (%)	94.3 (3.6)	94.3 (3.6)	94.1 (3.7)	0.993
Operated side (right/left)	Right: 31/Left: 23	15/14	16/9	0.526

Data are presented as mean (standard deviation) for continuous variables and as counts for categorical variables. Continuous variables were compared with independent-samples t-tests or Mann–Whitney U tests according to the Shapiro–Wilk test of normality; categorical variables were compared with chi-square tests. Stride length denotes the distance between two successive contacts of the same foot. Abbreviations: 2MWT, 2-Minute Walk Test; BMI, body mass index; FB, fixed-bearing; MB/PS-RP, mobile-bearing/posterior-stabilised rotating-platform; WOMAC, Western Ontario and McMaster Universities Osteoarthritis Index.

**Table 2:** Descriptive statistics for all outcomes at each postoperative assessment, stratified by prosthesis type.

Outcome	Assessment	FB, Mean (SD)	MB/PS-RP, Mean (SD)
2MWT distance (m)	First (T1)	75.16 (20.72) [*n* = 29]	73.01 (17.44) [*n* = 25]
	Second (T2)	89.08 (17.42) [*n* = 18]	86.96 (23.66) [*n* = 18]
	Third (T3)	64.17 (22.95) [*n* = 6]	84.3 (21.47) [*n* = 9]
Gait speed (m/s)	First (T1)	0.865 (0.271) [*n* = 29]	0.824 (0.223) [*n* = 25]
	Second (T2)	0.91 (0.201) [*n* = 18]	0.918 (0.263) [*n* = 18]
	Third (T3)	0.647 (0.253) [*n* = 6]	0.867 (0.246) [*n* = 9]
Cadence (steps/min)	First (T1)	98.2 (11.72) [*n* = 29]	98.65 (11.43) [*n* = 25]
	Second (T2)	100.43 (9.71) [*n* = 18]	103.71 (10.84) [*n* = 18]
	Third (T3)	92.0 (12.08) [*n* = 6]	100.79 (9.39) [*n* = 9]
Stride length (m)	First (T1)	1.057 (0.239) [*n* = 29]	1.009 (0.213) [*n* = 25]
	Second (T2)	1.104 (0.199) [*n* = 17]	1.066 (0.229) [*n* = 18]
	Third (T3)	0.842 (0.227) [*n* = 6]	1.031 (0.252) [*n* = 8]
Step-time symmetry index (%)	First (T1)	90.09 (8.86) [*n* = 29]	89.64 (8.39) [*n* = 25]
	Second (T2)	89.63 (8.89) [*n* = 18]	91.65 (7.9) [*n* = 18]
	Third (T3)	81.92 (13.28) [*n* = 6]	85.14 (13.53) [*n* = 9]
Gait quality index (%)	First (T1)	94.34 (3.58) [*n* = 29]	94.14 (3.71) [*n* = 25]
	Second (T2)	94.51 (3.27) [*n* = 18]	94.6 (1.96) [*n* = 18]
	Third (T3)	89.97 (9.86) [*n* = 6]	92.32 (3.32) [*n* = 9]

Data are presented as mean (standard deviation) with the number of contributing observations in square brackets. Abbreviations: 2MWT, 2-Minute Walk Test; FB, fixed-bearing; MB/PS-RP, mobile-bearing/posterior-stabilised rotating-platform; SD, standard deviation.

**Table 3:** Linear mixed-effects models assessing the effect of prosthesis type and time on functional and gait outcomes, with a random intercept per patient and adjustment for age and sex.

Outcome	Fixed Effect	Coefficient	95% CI	*p*-Value
Gait speed (m/s)	Prosthesis type (MB/PS-RP vs. FB)	−0.043	−0.162 to 0.076	0.478
	Time (per assessment interval)	−0.046	−0.126 to 0.034	0.262
	Time x prosthesis type	0.076	−0.032 to 0.184	0.168
Cadence (steps/min)	Prosthesis type (MB/PS-RP vs. FB)	0.405	−5.272 to 6.083	0.889
	Time (per assessment interval)	−0.815	−4.340 to 2.711	0.651
	Time x prosthesis type	2.554	−2.196 to 7.303	0.292
Stride length (m)	Prosthesis type (MB/PS-RP vs. FB)	−0.053	−0.161 to 0.055	0.334
	Time (per assessment interval)	−0.052	−0.122 to 0.018	0.149
	Time x prosthesis type	0.069	−0.027 to 0.165	0.158
Step-time symmetry index (%)	Prosthesis type (MB/PS-RP vs. FB)	−0.256	−5.174 to 4.663	0.919
	Time (per assessment interval)	−2.490	−5.856 to 0.876	0.147
	Time x prosthesis type	1.572	−2.951 to 6.095	0.496
Gait quality index (%)	Prosthesis type (MB/PS-RP vs. FB)	−0.291	−2.317 to 1.735	0.778
	Time (per assessment interval)	−1.348	−2.922 to 0.225	0.093
	Time x prosthesis type	0.705	−1.433 to 2.843	0.518
2MWT distance (m)	Prosthesis type (MB/PS-RP vs. FB)	−2.857	−13.082 to 7.369	0.584
	Time (per assessment interval)	1.730	−5.636 to 9.096	0.645
	Time x prosthesis type	4.887	−5.078 to 14.851	0.336

Coefficients are unstandardised. Prosthesis type is coded with FB as the reference category, so a positive coefficient indicates a higher value in the MB/PS-RP group. Time is expressed per assessment interval (approximately 12 months). Models were fitted on 105 observations from 54 patients, except stride length (103 observations from 54 patients, two observations having no recorded value). Abbreviations: CI, confidence interval; FB, fixed-bearing; MB/PS-RP, mobile-bearing/posterior-stabilised rotating-platform.

**Table 4:** Estimated marginal means with 95% confidence intervals at each postoperative assessment, derived from the mixed-effects models with time as a categorical factor and adjustment for age and sex.

Outcome	Assessment	FB, Marginal Mean (95% CI)	MB/PS-RP, Marginal Mean (95% CI)
2MWT distance (m)	First (T1)	75.1 (68.1 to 82.0)	73.1 (65.6 to 80.6)
	Second (T2)	88.7 (80.0 to 97.3)	87.0 (78.2 to 95.7)
	Third (T3)	66.3 (51.6 to 80.9)	82.3 (70.3 to 94.4)
Gait speed (m/s)	First (T1)	0.86 (0.78 to 0.95)	0.82 (0.74 to 0.91)
	Second (T2)	0.90 (0.80 to 1.01)	0.91 (0.81 to 1.02)
	Third (T3)	0.68 (0.51 to 0.85)	0.85 (0.71 to 0.99)
Cadence (steps/min)	First (T1)	98.1 (94.1 to 102.0)	98.5 (94.3 to 102.8)
	Second (T2)	101.0 (96.2 to 105.8)	103.2 (98.4 to 108.1)
	Third (T3)	92.4 (84.8 to 100.1)	100.3 (93.9 to 106.7)
Stride length (m)	First (T1)	1.06 (0.98 to 1.13)	1.01 (0.93 to 1.09)
	Second (T2)	1.09 (0.99 to 1.18)	1.06 (0.97 to 1.16)
	Third (T3)	0.87 (0.72 to 1.02)	1.02 (0.89 to 1.15)
Step-time symmetry index (%)	First (T1)	90.1 (86.7 to 93.5)	89.7 (86.0 to 93.3)
	Second (T2)	90.2 (85.9 to 94.5)	91.8 (87.5 to 96.1)
	Third (T3)	82.5 (75.3 to 89.6)	86.2 (80.2 to 92.1)
Gait quality index (%)	First (T1)	94.3 (92.9 to 95.8)	94.1 (92.6 to 95.7)
	Second (T2)	94.5 (92.7 to 96.4)	94.5 (92.7 to 96.4)
	Third (T3)	90.2 (87.0 to 93.3)	92.3 (89.8 to 94.9)

Marginal means were evaluated at the observed mean age and sex distribution of the analysed sample. Abbreviations: 2MWT, 2-Minute Walk Test; CI, confidence interval; FB, fixed-bearing; MB/PS-RP, mobile-bearing/posterior-stabilised rotating-platform.

**Table 5:** Between-group contrasts at each postoperative assessment, derived from the mixed-effects models with time as a categorical factor and adjustment for age and sex.

Outcome	Assessment	Difference (MB/PS-RP − FB)	95% CI	*p*-Value
2MWT distance (m)	First (T1)	−1.9	−12.1 to 8.3	0.712
	Second (T2)	−1.7	−14.0 to 10.6	0.788
	Third (T3)	16.1	−2.8 to 35.0	0.096
Gait speed (m/s)	First (T1)	−0.04	−0.16 to 0.08	0.537
	Second (T2)	0.01	−0.14 to 0.15	0.905
	Third (T3)	0.17	−0.05 to 0.39	0.123
Cadence (steps/min)	First (T1)	0.5	−5.3 to 6.3	0.869
	Second (T2)	2.3	−4.5 to 9.0	0.514
	Third (T3)	7.9	−2.1 to 17.8	0.122
Stride length (m)	First (T1)	−0.04	−0.15 to 0.06	0.424
	Second (T2)	−0.02	−0.15 to 0.11	0.740
	Third (T3)	0.15	−0.05 to 0.35	0.139
Step-time symmetry index (%)	First (T1)	−0.5	−5.5 to 4.6	0.860
	Second (T2)	1.6	−4.5 to 7.7	0.605
	Third (T3)	3.7	−5.5 to 13.0	0.431
Gait quality index (%)	First (T1)	−0.2	−2.3 to 1.9	0.855
	Second (T2)	−0.0	−2.6 to 2.6	0.999
	Third (T3)	2.2	−1.9 to 6.3	0.294

A positive difference indicates a higher value in the MB/PS-RP group. No adjustment for multiple comparisons was applied; contrasts are reported with their confidence intervals as prespecified descriptive quantities. Abbreviations: 2MWT, 2-Minute Walk Test; CI, confidence interval; FB, fixed-bearing; MB/PS-RP, mobile-bearing/posterior-stabilised rotating-platform.

**Table 6:** Within-group contrasts between postoperative assessments, derived from the mixed-effects models with time as a categorical factor and adjustment for age and sex.

Outcome	Group	Contrast	Estimate	95% CI	*p*-Value
2MWT distance (m)	FB	Second vs. first	13.6	3.8 to 23.4	0.007
	FB	Third vs. second	−22.4	−38.3 to −6.5	0.006
	FB	Third vs. first	−8.8	−24.1 to 6.5	0.260
	MB/PS-RP	Second vs. first	13.8	3.8 to 23.8	0.007
	MB/PS-RP	Third vs. second	−4.6	−18.3 to 9.1	0.507
	MB/PS-RP	Third vs. first	9.2	−3.8 to 22.2	0.166
Gait speed (m/s)	FB	Second vs. first	0.04	−0.07 to 0.15	0.466
	FB	Third vs. second	−0.23	−0.41 to −0.04	0.016
	FB	Third vs. first	−0.18	−0.36 to −0.01	0.041
	MB/PS-RP	Second vs. first	0.09	−0.03 to 0.20	0.127
	MB/PS-RP	Third vs. second	−0.06	−0.22 to 0.10	0.444
	MB/PS-RP	Third vs. first	0.03	−0.12 to 0.18	0.717
Cadence (steps/min)	FB	Second vs. first	2.9	−2.0 to 7.8	0.239
	FB	Third vs. second	−8.6	−16.6 to −0.6	0.036
	FB	Third vs. first	−5.6	−13.4 to 2.1	0.153
	MB/PS-RP	Second vs. first	4.7	−0.2 to 9.6	0.062
	MB/PS-RP	Third vs. second	−3.0	−9.8 to 3.9	0.397
	MB/PS-RP	Third vs. first	1.7	−4.8 to 8.3	0.598
Stride length (m)	FB	Second vs. first	0.03	−0.07 to 0.13	0.552
	FB	Third vs. second	−0.22	−0.38 to −0.05	0.009
	FB	Third vs. first	−0.19	−0.34 to −0.03	0.019
	MB/PS-RP	Second vs. first	0.05	−0.05 to 0.15	0.300
	MB/PS-RP	Third vs. second	−0.04	−0.19 to 0.10	0.561
	MB/PS-RP	Third vs. first	0.01	−0.13 to 0.15	0.887
Step-time symmetry index (%)	FB	Second vs. first	0.1	−4.7 to 4.9	0.961
	FB	Third vs. second	−7.8	−15.6 to 0.0	0.050
	FB	Third vs. first	−7.6	−15.2 to −0.1	0.046
	MB/PS-RP	Second vs. first	2.2	−2.7 to 7.0	0.383
	MB/PS-RP	Third vs. second	−5.6	−12.3 to 1.1	0.099
	MB/PS-RP	Third vs. first	−3.5	−9.9 to 2.9	0.286
Gait quality index (%)	FB	Second vs. first	0.2	−2.1 to 2.5	0.851
	FB	Third vs. second	−4.4	−8.0 to −0.7	0.018
	FB	Third vs. first	−4.2	−7.7 to −0.7	0.019
	MB/PS-RP	Second vs. first	0.4	−2.0 to 2.8	0.732
	MB/PS-RP	Third vs. second	−2.2	−5.4 to 1.0	0.172
	MB/PS-RP	Third vs. first	−1.8	−4.8 to 1.2	0.245

A positive estimate indicates a higher value at the later assessment. Contrasts involving the third assessment are based on six FB and nine MB/PS-RP patients and are correspondingly imprecise. No adjustment for multiple comparisons was applied. Abbreviations: 2MWT, 2-Minute Walk Test; CI, confidence interval; FB, fixed-bearing; MB/PS-RP, mobile-bearing/posterior-stabilised rotating-platform.

## Data Availability

The anonymised dataset analysed in this study, comprising 105 observations from 54 patients, is available from the corresponding author on reasonable request. The data are not publicly deposited because the ethics approval under which they were collected does not provide for public release of individual-level records.

## Abbreviations

The following abbreviations are used in this manuscript:

2MWT	2-Minute Walk Test
BMI	Body mass index
CI	Confidence interval
FB	Fixed-bearing
IMU	Inertial measurement unit
MB	Mobile-bearing
MB/PS-RP	Mobile-bearing/posterior-stabilised rotating-platform
OA	Osteoarthritis
SD	Standard deviation
STROBE	Strengthening the Reporting of Observational Studies in Epidemiology
TKA	Total knee arthroplasty
WOMAC	Western Ontario and McMaster Universities Osteoarthritis Index
